# Impact of end stage renal disease on the clinical outcomes of diabetics admitted for heart failure: Analysis of national inpatient sample

**DOI:** 10.34172/jcvtr.2023.30566

**Published:** 2023-03-16

**Authors:** Muhammad Usman Almani, Yaqi Zhang, Muhammed Hamza Arshad, Muhammad Usman, Muhammad Talha Ayub

**Affiliations:** ^1^Division of Cardiology, Albert Einstein Medical Center, Philadelphia, PA, USA; ^2^Division of Internal Medicine, John H. Stroger Hospital of Cook County, Chicago, IL, USA; ^3^Division of Internal Medicine, Waterbury Hospital, Waterbury, CT, USA; ^4^Division of Hospital Medicine, University of Wisconsin, Madison, WI, USA; ^5^Divsion of Cardiology, UPMC Heart and Vascular Institute, Pittsburgh, PA, USA

**Keywords:** End Stage Renal Disease, Diabetes Mellitus, Heart Failure

## Abstract

***Introduction:*** Patients with diabetes and heart failure (HF) can have varying outcomes depending on whether they also have End Stage Renal Disease (ESRD). This study aimed to compare the outcomes of patients with diabetes and HF with and without ESRD.

***Methods:*** Data from the National Inpatient Sample (NIS) 2016-2018 was analyzed to find hospitalizations for patients with HF as the main diagnosis and diabetes as a secondary diagnosis, with and without ESRD. Multivariable logistic and linear regression analysis was used to adjust for confounding factors.

***Results:*** In the total cohort of 12215 patients with a principal diagnosis of heart failure and secondary diagnosis of type 2 diabetes, the in-hospital mortality rate was 2.5%. Patients with ESRD had higher odds of in-hospital mortality (1.37x) compared to those without ESRD. The mean difference in length of stay was higher for patients with ESRD (0.49 days) and in total hospital charges (13360 US$). Patients with ESRD had higher odds of developing acute pulmonary edema, cardiac arrest, and requiring endotracheal intubation. However, they had lower odds of developing cardiogenic shock or requiring an intra-aortic balloon pump insertion.

***Conclusion:*** The results suggest that ESRD leads to higher in-patient mortality, length of stay, and total hospital charges for patients with diabetes admitted for HF. The lower incidence of cardiogenic shock and intra-aortic balloon pump insertion in patients with ESRD may be due to timely dialysis.

## Introduction

 Diabetes mellitus is an established risk factor for the development of heart failure (HF) and end stage renal disease (ESRD).^1,2^ It also leads to higher incidence of HF hospitalizations.^3^ The heart and kidneys interact in a bidirectional manner such that the declining glomerular filtration rate (eGFR) accelerates HF progression^4^ and intercurrent cardiovascular events, particularly heart failure, are strongly associated with increased risk for ESRD.^5^ Thus, diabetes mellitus, heart failure and ESRD often co-exist. Prior studies suggest that among patients with HF, cardiovascular mortality is elevated in patients with ESRD, and/or diabetes mellitus.^6^ However, there is limited data on mortality of HF patients with both diabetes mellitus and ESRD. We also analyzed less-studied clinical outcomes in diabetic patients hospitalized for HF such as incidence of cardiogenic shock, cardiac arrest, respiratory arrest requiring endotracheal intubation, and intra-aortic balloon pump (IABP), implantable cardioverter defibrillator (ICD) and pacemaker insertion.

 The purpose of this study is to compare and contrast the outcomes of diabetic patients hospitalized with a primary diagnosis of HF with a secondary diagnosis of ESRD to those who do not have ESRD. In CKD patients, recurrent interim HF was associated with an increased risk of mortality as well as a stepwise increase in the risk of developing ESRD.^7^ We hypothesize that the rate of negative outcomes including mortality, hospital stay, total hospital charge and the rates of cardiogenic shock, acute pulmonary edema and endotracheal intubation in patients with HF and ESRD to be higher compared to those without ESRD.

## Materials and Methods

###  Data Source

 We conducted a retrospective study of hospitalizations in 2016, 2017 and 2018 with a principal diagnosis of HF with secondary diagnosis of type 2 diabetes mellitus with and without a secondary diagnosis of ESRD in acute-care hospitals across the United States. Hospitalizations were selected from the NIS database (online at http://www.hcup-us.ahrq.gov). The National Inpatient Sample (NIS) is a large, publicly available database that contains information on hospital inpatient stays in the United States. It is maintained by the Healthcare Cost and Utilization Project (HCUP) and is sponsored by the Agency for Healthcare Research and Quality (AHRQ). This database contains information on demographic characteristics, diagnoses, procedures, charges, and outcomes for a representative sample of inpatient stays from hospitals across the United States. It is the largest all-payer inpatient care database in the United States. The 2016, 2017 and 2018 NIS sampling frame includes data from 47 states covering more than 97% of the U.S. population. As many as 30 discharge diagnoses for each hospitalization were recorded using the International Classification of Diseases, Tenth Revision, Clinical Modification (ICD-10) in NIS 2016 and 40 discharge diagnoses in NIS 2017 and 2018 database. Each hospital stay in the NIS is associated with one primary diagnosis, which is the main reason for the patient’s hospitalization, and one or more secondary diagnoses, which are additional conditions or complications that the patient has that are related to the primary diagnosis. The primary diagnosis is identified using the first-listed diagnosis field in the NIS data, while secondary diagnoses are identified using the other diagnosis fields. Since the NIS data is de-identified and publicly available, institutional review board approval was not sought for this study.

###  Inclusion Criteria and study variables

 We used data from the National Inpatient Sample (NIS) for the years 2016, 2017, and 2018 to analyze inpatient hospitalizations. The study variables included demographic characteristics such as age, gender, and race of the patients, as well as characteristics of the hospital where the patients were hospitalized, medical comorbidities, and primary and secondary outcomes (outlined below). We used the following ICD-10 codes to identify principal/secondary diagnoses: HF I50.x, Type 2 diabetes mellitus E11.x, ESRD N18.6. We studied baseline characteristics and outcomes for heart failure hospitalization with type 2 diabetes mellitus with and without ESRD.

###  Outcomes

 The primary outcome was comparing inpatient mortality among diabetic patients principally admitted for heart failure with and without secondary diagnosis of end stage renal disease. Hospital length of stay (LOS), total hospital charge, odds of cardiogenic shock, intra-aortic balloon pump insertion, implantable cardioverter defibrillator insertion, pacemaker insertion, cardiac arrest and endotracheal intubation were secondary outcomes of interest.

###  Statistical Analysis

 STATA software (version 16) was used to perform the statistical analyses. The student t-test and the chi-squared test were used to compare continuous and categorical variables, respectively. Multivariable Logistic and Linear regression was used to estimate odds ratios and adjusted mean difference for the primary and secondary outcomes. All the outcomes were adjusted for patient level and hospital level covariates.

## Results

 The National Inpatient Sample (NIS) database for the years 2016-2018 contains a large amount of data, including over 107 million weighted hospital discharges. Out of those, we included 488,140 hospital discharges that satisfied our inclusion criteria. These patients were adults (age > 18 years) with a principal discharge diagnosis of heart failure and a secondary diagnosis of type 2 diabetes mellitus. 6.5% (n = 31870) of these patients had an additional secondary diagnosis of end stage renal disease (ESRD), defined by ICD-10 codes.

 Patients with comorbid ESRD were significantly younger (mean age: 67 years vs. 71 years, *P* < 0.001) at the time of hospital admission compared to the patients without ESRD. The racial distribution was significant with more Black patients (30% vs. 19.1%) and Hispanic patients (16.5% vs. 9.1%) in the ESRD group. Patients with comorbid ESRD had lower median annual income and higher Charlson comorbidity index as depicted in Table 1. Patients in the ESRD group were more likely to have anemia (73.1% vs. 33.5%, *P* < 0.001) and peripheral vascular disease (13.7% vs. 8.8%, *P* < 0.001) however, they were less likely to have smoking history (26.2% vs. 29.6%, *P* < 0.001), chronic obstructive pulmonary disease (32.2% vs. 37.6%, *P* < 0.001) and presence of aorto-coronary bypass graft (16.2% vs. 18%, *P* < 0.001). The in-hospital mortality was 2.5% (n = 12215) of the total cohort of patients who had a principal discharge diagnosis of heart failure with a secondary diagnosis of type 2 diabetes mellitus. Additional patient and hospital characteristics are shown in Table 1.

**Table 1:** Patient and hospital characteristics of diabetic patients admitted for heart failure based on presence or absence of ESRD, in the US from 2016-2018.

**Variable**	**Overall %**	**Without ESRD %**	**With ESRD %**	***P*** ** value**
		n = 456,270 (93.5)	n = 31870 (6.5)	
Patient characteristics				
Age (years), mean		71	67	< 0.001
Women	47.4	47.4	48.2	0.222
Racial distribution				< 0.001
White	65.1	66.6	44.4	
Black	19.8	19.1	30.0	
Hispanic	9.6	9.1	16.5	
Others	5.5	5.3	9.1	
Insurance type				< 0.001
Medicare	73.4	73.1	77.4	
Medicaid	10.6	10.6	10.6	
Private	11.7	11.9	9.2	
Self-pay	2.2	2.3	1.3	
Other	2.2	2.2	1.6	
Charlson Comorbidity Index score, no				< 0.001
0-5	69.9	71.5	46.5	
6-10	29.6	28.0	52.7	
> 10	0.5	0.5	0.9	
Median annual income				< 0.001
1-43,999	36.4	36.1	39.4	
44,000-55,999	27.1	27.2	26.1	
56,000-73,999	21.8	21.9	20.8	
≥ 74,000	14.8	14.8	13.7	
Co-morbidities				
Smoking history	29.4	29.6	26.2	< 0.001
Dyslipidemia	56.8	56.9	55.5	0.034
Anemia	36.1	33.5	73.1	< 0.001
Obesity	31.0	31.6	21.8	< 0.001
PVD	0.9	8.8	13.7	< 0.001
Hypothyroidism	17.8	17.9	16.5	0.007
Hypertension	32.8	34.9	1.8	< 0.001
Liver disease	5.3	5.3	5.3	0.867
COPD	37.3	37.6	32.2	< 0.001
Carotid artery disease	1.4	1.4	1.1	0.130
Old MI	16.4	16.4	16.6	0.742
Coronary angioplasty status	2.06	2.04	2.34	0.106
Presence of aorto-coronary bypass graft	17.9	18.0	16.2	< 0.001
Coronary artery disease	57.6	57.4	60.6	< 0.001
Renal artery stenosis	0.4	0.4	0.4	0.662
Hospital characteristics				
Hospital region				< 0.001
Northeast	18.5	18.5	18.2	
Midwest	23.5	23.7	20.3	
South	40.6	40.6	40.5	
West	17.4	17.2	21.0	
Hospital bed size, no. (%)				< 0.001
Small	22.1	22.5	17.1	
Medium	28.9	28.7	30.6	
Large	49.0	48.8	52.3	
Hospital location and teaching status				< 0.001
Rural	14.7	15.2	7.1	
Urban nonteaching	26.8	26.6	28.7	
Urban teaching	58.6	58.2	64.2	

Abbreviation: PVD, peripheral vascular disease; COPD, chronic obstructive pulmonary disease; MI, myocardial infarction

 Patients with comorbid ESRD had higher odds of in-hospital mortality (aOR: 1.37, 95% CI 1.18 – 1.59, *P* < 0.001) when adjusted for patient and hospital factors and co-morbidities. The total length of hospitalization and the total hospital charges between the groups were compared using a multivariate linear regression model. Patients with ESRD had a higher mean difference in length of stay (adjusted mean difference in days: 0.49, 95% CI 0.29 – 0.70, *P* < 0.001) as well as higher mean difference in total hospital charges (adjusted mean difference in US$: 13360, 95% CI 9824 – 16898, *P* < 0.001). Patients in the ESRD group had higher odds of developing acute pulmonary edema (aOR: 3.15, 95% CI 2.50 – 3.97, *P* < 0.001), cardiac arrest (aOR: 1.85, 95% CI 1.43 – 2.41, *P* < 0.001) and having endotracheal intubation (aOR 1.35, 95% CI 1.14 – 1.61, p = 0.001). ESRD patients were less likely to develop cardiogenic shock (aOR: 0.57, 95% CI 0.46 – 0.71, *P* < 0.001) or have intra-aortic balloon pump insertion (aOR: 0.28, 95% CI 0.12 – 0.64, *P* = 0.003). Detailed clinical outcomes are listed in Table 2.

**Table 2:** Clinical outcomes of patients with type 2 diabetes mellitus admitted for heart failure with and without co-existing end stage renal disease (ESRD) in the U.S from 2016 through 2018; analysis of inpatient sample

**Outcome**	**Without ESRD; %**	**With ESRD; %**	**aOR (95% CI)**	***P*** value*
Primary outcome				
In hospital mortality	2.4	3.5	1.37 (1.18 – 1.59)	< 0.001*
Secondary outcomes				
Length of stay (days); mean	5.3	6.3	0.49 (0.29 – 0.70) ^#^	< 0.001*
Total hospital charges (US$); mean	47551	69015	13360 (9824 – 16898) ^#^	< 0.001*
Acute pulmonary edema	0.5	1.8	3.15 (2.50 – 3.97)	< 0.001*
Cardiogenic shock	1.9	1.5	0.57 (0.46 – 0.71)	< 0.001*
IABP insertion	0.3	0.1	0.28 (0.12 – 0.64)	0.003*
ICD insertion	0.5	0.5	0.92 (0.62 – 1.36)	0.671
Pacemaker insertion	0.4	0.5	1.26 (0.86 – 1.86)	0.241
Cardiac arrest	0.6	1.2	1.85 (1.43 – 2.41)	< 0.001*
Endotracheal intubation	1.5	2.6	1.35 (1.14 – 1.61)	0.001*

Abbreviations: aOR, adjusted odds ratio; CI, confidence interval; IABP, Intra-aortic balloon pump; ICD, implantable cardioverter defibrillator Adjusting factors, age; sex; race; Charlson comorbidity index; median household income; hospital location and teaching status; dyslipidemia; hypertension; obesity; peripheral vascular disease; liver disease; smoking; coronary angioplasty status and old myocardial infarction ^*^statistically significant; ^#^adjusted mean difference

## Discussion

 A significant proportion of patients with HF have underlying DM and renal dysfunction. Heart failure (HF), diabetes mellitus (DM) and chronic kidney disease (CKD) often coexist in certain patient population, and studies have shown diabetic patients are much more likely to develop HF.^8^ Prevalence of diabetes in patients with HF is as high as approximately 28%,^9^ the incident risk of HF increases with the severity of CKD.^10^ In our study, we queried the diabetic patients who were admitted for HF during 2016-2018 from NIS database, and the number of hospitalizations is almost half a million (n = 488,140). End stage renal disease (ESRD) was co-morbid secondary diagnosis in 6.5% of this population, which is in line with previous studies showing incidence of HF is 7% per year in dialysis patients, about 12-36 times more common than the general population.^11^

 Declining estimated glomerular filtration rate (eGFR) accelerates heart failure progression and heart failure increases the risk for end stage renal disease.^4,5^ In our analysis, there were more Black (30% vs. 19.1%) and Hispanic patients (16% vs. 9.1%) with ESRD than without ESRD. Also, the patients with ESRD were younger (67 years vs. 71 years, *P* < 0.001) at the time of hospital admission for heart failure. In diabetic patients with chronic kidney disease, progression to ESRD is demonstrated to be higher among Black and Hispanic patients compared to the White patients.^12^ Among patients who develop ESRD, the decline in eGFR is greater than the patients who do not develop ESRD.^12^

 Studies have shown that CKD is a risk multiplier for cardiovascular morbidity, mortality, heath care utilization and costs.^13,14,15^ Similarly, diabetic patients with ESRD who were admitted for HF in our study had higher hospital mortality (aOR: 1.37, 95% CI 1.18 – 1.59, *P* < 0.001), longer hospital stay (adjusted mean difference in days: 0.49, 95% CI 0.29 – 0.70, *P* < 0.001) and had posed higher financial burden on the health care system (adjusted mean difference in USD: 13360, 95% CI 9824 – 16898, *P* < 0.001). Furthermore, we included several specific secondary outcomes in the study patient population, which were imperfectly investigated in previous studies. Cardiogenic shock is not a frequent clinical event in HF hospitalizations but relates to substantial mortality.^16,17^ Previous study using NIS data showed non-ACS-related cardiogenic shock had an incidence rate of 1.5% among HF hospitalizations,^16^ our study also showed similar incidence rate among diabetic patients admitted for HF. Interestingly, we found that patients with ESRD had a lower incidence rate of cardiogenic shock (aOR: 0.57, 95% CI 0.46 – 0.71, *P* < 0.001). Volume overload is an independent predictor of mortality in dialysis patients.^18^ Diabetes mellitus and pre-existing cardiovascular disease relates to overhydration in the ESRD patients. ^18^ Timely and scheduled renal replacement therapy for ESRD patients prevents consequences like volume overload and electrolyte disturbances and could explain the lower incidence of cardiogenic shock in this population. We also investigated short-term mechanical circulatory support and synchronizing device use in studied population, including IABP, ICD and pacemaker insertion rates. Only IABP insertion rate had a statistically significant difference between two groups, with a lower application rate in patients with ESRD (aOR: 0.28, 95% CI 0.12 – 0.64, *P* = 0.003). The result corresponds to the differences between incidence rates of cardiogenic shock. Some studies demonstrated that IABP support could stabilize patients with cardiogenic shock or advanced HF, to earn additional time for bridging to durable ventricular assist devices (VADs) or heart transplant.^19,20^ Short-term circulatory support can be a reasonable choice in diabetic patients with renal dysfunction and advanced heart failure, to earn additional time to proceed with durable treatments. Cardiac arrest is a known devastating complication in people with ESRD undergoing dialysis.^21,22^ Our study also concludes that diabetic patients in HF hospitalization are more prone to end in cardiac arrest if complicated by ESRD (aOR: 1.85, 95% CI 1.43 – 2.41, *P* < 0.001).

 Cardiovascular disease is a major cause of pulmonary edema in CKD patients, especially those requiring chronic dialysis.^23,24^ We found that the admissions we investigated were more likely to be complicated by acute pulmonary edema if the patients had ESRD (aOR: 3.15, 95% CI 2.50 – 3.97, *P* < 0.001). Death rates of acute pulmonary edema in patients with CKD or HF varies in different studies.^24,25^ HF complicated by acute pulmonary edema is one of the major factors which increase the hospital admission rate for ESRD patients,^26^ and the dramatic presentation usually results in ICU stay.^24^ In the meanwhile, advanced respiratory support like endotracheal intubation rate is much higher in patients with ESRD from our study (aOR: 1.35, 95% CI 1.14 – 1.61, *P* = 0.001). Thus, pulmonary presentations for diabetic patients with renal dysfunction who present with HF should be paid more attention in order to reduce step-up hospital care.

 Recent trials show efficacy of SGLT-2 inhibitors in reducing the hospitalization for heart failure and progression of renal disease especially in diabetic patients ^27,28^ and sacubtril-valsartan improves outcomes of heart failure in patients with ESRD.^1^ This presents promising area for future studies in terms of comparing the outcomes of treatment modalities for heart failure in diabetics with kidney disease.

 Several limitations apply to this study. Firstly, NIS database studies are subject to all the biases associated with retrospective studies including selection bias, confounding bias and measurement bias. Second, the NIS data is based on administrative claims data, which may not always be accurate or complete. There may be errors or missing data in the coding of diagnoses or procedures, which could affect the results of the study. Third, since the database does not include detailed information on patients, therefore, individuals hospitalized multiple times with the same principal discharge diagnosis would be counted multiple times. Fourth, it does not include information on the cause of inpatient mortality. Lastly, it does not include certain data that can provide a more comprehensive understanding of the patient’s condition such as disease duration, treatment received, extent of disease, laboratory results and long-term mortality.

## Conclusion

 ESRD leads to higher in-patient mortality, higher length of stay and higher total hospital Charges in type 2 diabetics admitted for HF. Incidence of cardiogenic shock and intra-aortic balloon pump insertion is less in ESRD patients, likely due to timely interval dialysis leading to hemodynamic and electrolyte homeostasis.

## Acknowledgements

 None.

## Competing Interests

 The authors have no conflicts of interest to declare.

## Ethical Approval

 Data for this study was obtained from querying the Nationwide Inpatient Samples, which are publicly available datasets. Per 45 Code of Federal Regulations (CFR 46.101),

 research using certain publicly available data sets does not involve “human subjects”.

 The data contained within these specific data sets are neither identifiable nor private and thus do not meet the federal definition of “human subject” as defined in 45 CFR

 46.102. These research projects do not need to be reviewed and approved by the IRB.

## Funding

 Authors have no disclosures.
